# Morphoproteomic-Guided Host-Directed Therapy for Tuberculosis

**DOI:** 10.3389/fimmu.2017.00078

**Published:** 2017-02-02

**Authors:** Robert E. Brown, Robert L. Hunter, Shen-An Hwang

**Affiliations:** ^1^Department of Pathology and Laboratory Medicine, McGovern Medical School, University of Texas Health Science Center at Houston, Houston, TX, USA

**Keywords:** morphoproteomics, tuberculosis, host-directed therapy, mTOR, COX-2

## Abstract

In an effort to develop more effective therapy for tuberculosis (TB), research efforts are looking toward host-directed therapy, reprograming the body’s natural defenses to better control the infection. While significant progress is being made, the efforts are limited by lack of understanding of the pathology and pathogenesis of adult type TB disease. We have recently published evidence that the developing lesions in human lungs are focal endogenous lipid pneumonia that constitutes a region of local susceptibility in a person with strong systemic immunity. Since most such lesions regress spontaneously, the ability to study them directly with immunohistochemistry provides means to investigate why some progress to clinical disease while others asymptomatically regress. Furthermore, this should enable us to develop more effective host-directed therapies. Morphoproteomics has proven to be an effective means of characterizing protein expression that can be used to identify metabolic pathways, which can lead to more effective therapies. The purpose of this perspective will argue that using morphoproteomics on human TB lung tissue is a particularly promising method to direct selection of host-directed therapeutics.

Progression of pulmonary tuberculosis (TB) in adults is a rare phenomenon in that at least 90% of cases regress spontaneously without producing clinical disease. Very little is known of why and how infection progresses to clinical disease in some people despite spontaneously regression in most. What is known is that TB disease is multifaceted, involving not just the actions of the pathogen *Mycobacterium tuberculosis* (MTB) on the host but also various immune mechanisms in response to bacterial antigens. TB disease is a chronic infection in immune competent hosts, displaying different pathologies, often simultaneously, in microenvironments in the same infected tissue, mostly in the lung (1–3). Protection from and progression to TB disease involves similar immune responses (4–6), and ongoing studies are trying to tease apart these differences.

There is no question that host immune responses play crucial roles in disease progression and transmission, but currently no therapeutic has been developed to suppress the immune induced pathology. Such host-directed therapy is routinely used and invested heavily in research in cancer (7–12), autoimmune (13–15), inflammatory (16), and other immune based diseases. Recently, immune directed therapy has been proposed and demonstrated to be potentially effective in TB disease (17–20). In order for this therapy to be effective, correct identification of critical host immune targets is paramount. This paper discusses newly developed means of studying host responses important for progression of pulmonary TB disease.

Host-directed therapy targets pathological mechanisms, either by shutting down pathways or manipulating immune responses to improve protection against the MTB pathogen. Proper identification of these pathological targets is crucial for the effectiveness of any host-directed therapy. Many pathological mechanisms of TB overlap with other immune-based diseases, providing TB researchers with a vast foundation of commercially available drugs (17) that have demonstrated protective responses in TB models. The use of *in vitro* and *in vivo* models to tease apart mechanistic parameters of diseases may be useful but may not adequately represent the human disease. Thus, targets identified through TB models may not be effective in the human patient. The best method to select effective targets for host-directed therapy for TB disease is by studying the human patient.

*Mycobacterium tuberculosis* is an obligate human pathogen since only humans develop cavities able to expel large numbers of organisms into the environment to infect new hosts (21–24). In order to eliminate TB disease, MTB transmission must be stopped by attenuating the caseation pathology. One key feature of caseation is that it occurs in localized pulmonary sites. Most people retain a high level of immunity in every part of their bodies except in localized pulmonary lesions. These lesions are areas of localized susceptibility that coexists with systemic immunity. Understanding the host mechanisms at these localized lesions that lead to susceptibility of MTB infection is hampered by the lack of access to appropriate clinical samples.

Since human tissues have not been available to most investigators since the introduction of antibiotics in the 1950s current descriptions of human pulmonary TB are based on animal models. While there are many animal models of TB, none of them develop pulmonary TB like humans. Consequently, some features of the pathology of human pulmonary TB have been largely forgotten. Through an extended study of human tuberculous tissues and relevant literature, we have formulated a corrected understanding of the pathology of human pulmonary TB and a new paradigm of its pathogenesis, reviewed extensively elsewhere (22, 25). The key finding is that pulmonary TB has a prolonged period of asymptomatic infection of alveolar macrophages in particular parts of the lung before the onset of clinical disease. This results from a localized susceptibility in parts of a lung in an otherwise immune person. A better understanding of how and why most of these lesions regress, while others progress to clinical disease might suggest ways to make them all regress and thereby eliminate TB.

Currently, most clinical samples from TB patients are either blood or lung. The former examine responses and mechanisms that are systemic, not at the site of infection, and may represent secondary effects of the primary response in the lung tissue. Alveolar lavages and lung biopsies are limited in the information they can provide: lavages are limited to immune responses in the alveolar and biopsies samples are too small to incorporate the surrounding parenchyma. Our group has taken the approach that in order to understand TB pathology we must study lung samples taken from untreated or under-treated TB individuals. Successful antibiotic treatment is known to significantly alter pathology (26–30). As MTB is killed by the antibiotic, the antigens that stimulate the host immune responses that generate the lung pathology are cleared. We propose that autopsy samples obtained from these untreated individuals may be the key to understanding the mechanisms of TB disease pathogenesis, especially the caseation process. Correct identification of key factors engaged in the caseation process will allow design of therapy directed toward controlling and ultimately stopping the pathology and arresting transmission of MTB.

There are several methods to identify pathological factors in lung tissue of TB patients. Our strategy has always focused on protein expression, as RNA expression may not necessarily result in changes in protein level due to post translational regulation. Additionally, we do not think that global proteomics will be useful due to the nature of MTB microenvironments. Within a single patient, MTB infection creates microenvironments with varying degree of pathology. In our samples, we often observed, in a single tissue section, areas of lipid pneumonia, matured cavities, developing cavities, fibrosis, caseation, and normal lung parenchyma. Each of these microenvironments has a different profile of immune responses, thus global proteomics will be unable to tease apart the critical targets of caseation. Our group proposes that the best method to identify effective targets for host-directed therapy is through the use of morphoproteomics, analyzing protein expression profile of specific pathological microenvironments.

Morphoproteomics is defined “as the identification by immunohistochemistry of the molecular circuitry of various proteins…by noting their state of activation (translocation and phosphorylation) and correlative expressions” (31). The method was originally developed for cancer patients, as tumors are often heterogeneous and was hypothesized to be more responsive to individualized guided therapy (32) as opposed to generalized standard protocols. Since this proposed method was developed in 2004 by Robert Brown, several publications have been peer reviewed and reported as to its effectiveness. A search in PubMed has yielded 37 publications. The majority of these used morphoproteomics to identify potential targets for adjuvant host-directed therapy (33–43) for an extensive list of cancers, such as prostate cancer, head and neck squamous cell carcinoma, Kaposi’s sarcoma, Hodgkin lymphoma, and others. Several publications have also indicated the clinical effectiveness of using morphoproteomics to guide host-directed therapy using commercially available drugs, including glioblastoma (44), osteosarcoma (45), pediatric brain tumors (46), and others. The success of morphoproteomic-guided therapy in cancer indicates that this method can be applied to other diseases where there is heterogeneous pathology and the host response directly causes the disease pathogenesis. Though morphoproteomics, we are able to identify cell types and characterize pathways in isolated lesions in human lungs. This manuscript reports recent findings and suggests future studies to investigate this key aspect of TB that takes place only in human lungs.

We propose that the heterogeneity of TB disease and the critical roles that the host response plays in the disease pathogenesis strongly indicate that morphoproteomic-guided host-directed therapy can be an effective tool to identify drugs with high possibility of ameliorating TB induced pathology. We believe that the future of host-directed therapy is to verify that pathology mechanisms identified in *in vitro* and/or animal models do occur in the human disease but also to demonstrate that the selected target(s) will affect critical pathology. From our extensive studies of human TB pathology, we hypothesize that foamy alveolar macrophages (obstructive lipid pneumonia) are the critical pathology directly responsible for the development of cavities (25). Thus, modulation of these pathologic macrophages may affect progression of pathology, the eventual cavitation, and stop the transmission process. As an example of how morphoproteomic-guided host-directed therapy can be applied, we decided to focus initially on two mechanisms of how MTB controls the host macrophage responses to promote its survival: mammalian target of rapamycin (mTOR) and cyclooxygenase 2 (COX-2) pathways.

*Mycobacterium tuberculosis* has evolved to escape host cell killing by preventing phagosome maturation into an acidic vesicle, the phagolysosome. Recent discoveries found that activation of autophagy through inhibition of mTOR can stimulate a double-membrane autophagosome that is capable of killing intracellular MTB (47, 48). The mTOR protein can bind other proteins to form two distinct complexes: mTORC1 (raptor-associated) that is sensitive to rapamycin and mTORC2 (rictor-associated) that is insensitive to rapamycin. In the context of MTB infection (both mouse and human studies), only mTORC1 has shown to be associated with TB disease. Since then, several animal studies have investigated the effectiveness of using rapamycin to inhibit mTOR as adjunct therapy (49–51) or for vaccination (52, 53). We completed preliminary staining of a clinical lung sample from a female who died suddenly at home. Diagnosis of TB disease was done at autopsy. No history of TB treatment was noted. Pathological analysis demonstrated the presence of foamy macrophages in alveolar spaces. We chose to stain for three markers of mTOR signaling (Figure 1). Foamy macrophages are heavily positive for expression of activated, pmTOR, phosphorylated (p) on serine 2448. Additionally, pmTOR is also positive in the alveolar walls, but to a lesser intensity. The second marker examined is the expression of insulin-like growth factor-1 receptor (IGF-1R), a strong inducer of mTOR *via* PI3K. Presence of IGF-1R is expressed not only in foamy macrophages but also in the surrounding parenchyma. The third marker is activated Akt (pAkt, on serine 473), the putative downstream effector of mTORC2 (54–57). We observed minimal presence of pAkt in foamy macrophages, suggesting that during MTB infection foamy macrophages are overexpressing mTORC1 and little to no activation of mTORC2. Activation of mTORC1 causes a negative feedback that decreases pAkt (58). This preliminary study suggests that foamy macrophages in this MTB-infected lung tissue over activate mTORC1, inhibiting autophagy of the infected cell and limiting MTB killing.

**Figure 1:**
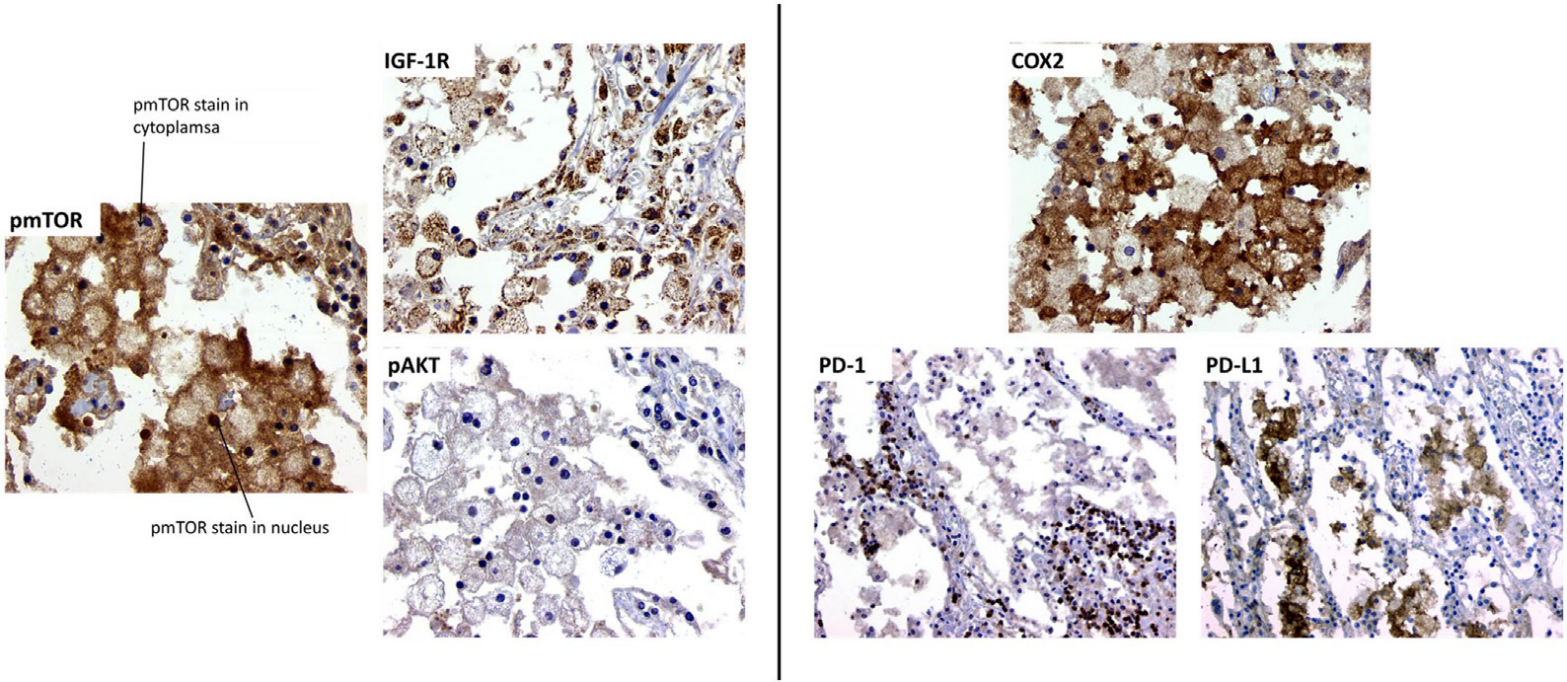
**Morphoproteomic analysis of human TB lung sample**. Left: stain for phosphorylated mTOR, insulin-like growth factor-1 receptor (IGF-1R), and phosphorylated Akt at 400× magnification. Right: sample stained with anti-human cyclooxygenase 2 (COX-2) and visualized at 400×. Programed death-1 (PD-1) and programed death-1 ligand (PD-L1) stain, magnification at 200×.

We also examined a second pathway of macrophage activity, COX-2. All studies of macrophage cultures suggest that MTB infection inhibit COX-2 activation and production of prostaglandin E2 (PGE2), leading to necrosis of the MTB-infected cell and MTB escape and spread of infection (59, 60). However, the effect of COX-2 activation in the *in vivo* lung local environment during MTB infection has not been well studied. Published reports on the cancer microenvironment often demonstrate that upregulation of COX-2 and PGE2 correlated to an increase in the presence and activity of T regulatory cells, which directly inhibited activity and function of effector T cells (61). Upregulation of T regulatory cells during active MTB infection blocks the ability of effector T cells to activate macrophages to control MTB infection (62), leading to loss of pathogen containment, uncontrolled proliferation, pathological inflammation, tissue necrosis, and spread of infection. Indeed, in the lungs of mice infected with MTB, COX-2 and PGE2 are overexpressed (63), suggesting that lung macrophage COX-2 activity may not reflect *in vitro* macrophage cultures studied. In this one MTB-infected lung sample, foamy macrophages varied in COX-2 intensity, indicating variability in the amount of COX-2 being expressed. Interestingly, COX-2 expression is mainly restricted to the foamy macrophage with nearly no COX-2 positivity in the alveolar walls (Figure 1).

In cancer studies, expression of COX-2 is associated with increase in T regulatory cells (64). T regulatory cell expansion in TB disease is associated with increases in expression of programed death-1 ligand (PD-L1) on antigen-presenting cells (65, 66). The expression of PD-L1 acts directly on programed death-1 (PD-1)-expressing T cells to inhibit their effector functions (67–70). In this MTB-infected lung microenvironment, PD-L1 is highly expressed in foamy macrophages, surrounded by PD-1-expressing lymphocytes in the interstitial (Figure 1). This suggests that foamy macrophages in this MTB-infected lung favor T effector cell suppression, possibly through macrophage COX-2 production. The increase in COX-2 in macrophages enhances surface expression of PD-L1, which ligates to PD-1, inhibiting activity of PD-1-expressing effector T cells. The increase in COX-2-producing macrophage may be due to increases in T regulatory cells in the MTB microenvironment, as previously observed (62). Thus, in this critical MTB microenvironment of foamy alveolar macrophages, two suppressor host response pathways are active (mTOR and COX-2), allowing TB disease progression.

Additional lung samples are currently undergoing the same morphoproteomic analysis. At the time of this manuscript preparation, four additional human TB lung samples have demonstrated the same pattern of mTOR and COX-2 staining in the alveolar macrophage pathological microenvironments (Hwang observations). With both mTOR and COX-2 mechanisms are potentially highly active in TB lungs, it seems logical to argue that designing host-directed therapy that would target both pathological pathways should offer the most success. There are several FDA-approved drugs that target mTOR and COX-2. Using commercially available products will enable clinical testing of the proposed therapy once proof-of-concept is established in appropriate animal models. We have identified two inhibitors that may offer the most effective outcome in reversing MTB-induced host pathological responses.

## mTOR Inhibitor

Metformin is an antidiabetic drug that activates adenosine monophosphate-activated kinase. *In vitro* analysis showed that Metformin directly reduced phosphorylation of mTOR and p70S6k, increasing apoptosis (71). Diabetic patients co-infected with MTB on metformin lived longer than those not taking metformin. *In vivo* mouse model studies demonstrated a significant decrease in lung bacterial load and pathology when treated with metformin (72). We believe metformin will inhibit overexpression of pmTOR and the expression of mTORC1 leading to decreased presence of foamy macrophages and increased autophagy and/or increased MTB-infected cell apoptosis, leading to observable decreases in bacterial load and lung pathology.

## COX-2 Inhibitor

Celecoxib is a non-steroid anti-inflammatory drug that is a COX-2 inhibitor. However, celecoxib is capable of blocking several other proteins in the COX-2 signaling pathway and antiapoptotic proteins, such as Bcl-2 and Mcl-1 (73). We believe that using celecoxib during MTB disease will enhance apoptosis of foamy macrophages and increase effector T cell function, leading to decreased bacterial load and lung pathology, as observed by decreased clusters of foamy macrophages.

This is only one example of how morphoprotemics can aid in selection of host-targeted therapy. While human lung tissues have been previously investigated by immunohistochemistry, all these studies focused on MTB proteins (usually antigens) and/or host immune cell surface/secreted proteins (62, 74–77). Morphoproteomics is capable of identifying cell signaling pathways that are active in respect to specific pathological microenvironments, enabling understanding of the immunometabolism mechanisms that may be attractive targets for host-directed therapy (78). The application of routine morphoproteomic analysis for TB disease is still in its infancy due to the lack of appropriate human TB lung tissue and knowledgeable clinical pathologists. We are making progress toward creating a human TB pathology consortium that other researchers may access. Enabling researchers to verify their findings in the human patient is a must if we are to make significant breakthroughs in the future of TB research.

We offer the promise of an alternative strategy to developing new treatments for TB beyond just searching for effective antibiotics or choosing host-directed therapeutic targets from *in vitro* and/or animal models. Our approach is unique in that morphoproteomics directly analyzes pathological mechanisms in human tissue, allowing selection of targets for therapy that have been proven to be correlated with human disease. Additionally, morphoproteomics can also be used to tailor host-directed therapy to the individual patient, as it has been applied in cancer patients (32, 34, 45), if necessary. Since foamy alveolar macrophages are the lesion of TB that frequently undergoes spontaneous regression, we believe that studying it with morphoproteomics will identify the most promising targets for clinical testing and offer the highest chance of a positive outcome, to reduce or eliminate MTB transmission and reduce progression of the disease to the cavitary formation.

## Author Contributions

S-AH prepared the manuscript and did the final data analysis. RB performed the morphoproteomic analysis and help edit the manuscript. RH provided the pathology analysis and edit the manuscript.

## Conflict of Interest Statement

The authors declare that the research was conducted in the absence of any commercial or financial relationships that could be construed as a potential conflict of interest. The reviewer IO and handling Editor declared their shared affiliation, and the handling Editor states that the process nevertheless met the standards of a fair and objective review.
